# Pre-frontal stimulation does not reliably increase reward responsiveness

**DOI:** 10.1016/j.cortex.2022.11.011

**Published:** 2023-02

**Authors:** L.M. Hadden, H. Penny, A.L. Jones, A.M. Partridge, T.M. Lancaster, C. Allen

**Affiliations:** a Cardiff University, School of Psychology, Tower Building, Park Place, Cardiff, CF10 3AT, UK; b School of Psychology, Faculty of Medicine, Health, and Life Sciences, Singleton Park, Swansea University, SA2 8PP, UK; c University of Sheffield, Research Services, New Spring House, 231 Glossop Road, Sheffield, S10 2GW, UK; d University of Bath, Department of Psychology, Claverton Down, BA2 7AY, UK; e Department of Psychology, Durham University, Durham, DH1 3LE, UK; f Aneurin Bevan University Health Board, St Cadoc's Hospital, Lodge Road, Caerleon, NP18 3XQ, UK

**Keywords:** Transcranial magnetic stimulation, Reward responsiveness, Anhedonia, Depression, Replication

## Abstract

Depression is the leading cause of disability worldwide and its effects can be fatal, with over 800,000 people dying by suicide each year. Neuromodulatory treatments such as transcranial magnetic stimulation (TMS) are being used to treat depression. Despite its endorsement by two regulatory bodies: NICE (2016) and the FDA (2008), there are major questions about the treatment efficacy and biological mechanisms of TMS. Ahn et al.’s (2013) justified the use of TMS in a clinical context in an important study indicating that excitatory TMS increases reward responsiveness. A pseudo-replication of this study by Duprat et al., (2016) also found a similar effect of active TMS, but only with the addition of an exploratory covariate to the analyses–trait reward responsiveness. Here we replicate Ahn et al.’s (2013) key study, and to test the reliability of the effects, and their dependency on trait reward responsiveness as described by Duprat et al., (2016). Using excitatory and sham TMS, we tested volunteers using the probabilistic learning task to measure their reward responsiveness both before and after stimulation. We also examined affect (positive, negative) following stimulation. Irrespective of TMS, the task was shown to be sensitive to reward responsiveness. However, we did not show TMS to be effective in increasing reward responsiveness and we did not replicate Ahn et al., (2013) or Duprat et al., (2016)'s key findings for TMS efficacy, where we provide evidence favouring the null. Moreover, exploratory analyses suggested following active stimulation, positive affect was reduced. Given our findings, we question the basic effects, which support the use of TMS for depression, particularly considering potential deleterious effects of reduced positive affect in patients with depression.

## 1 Introduction

Depression is the leading cause of disability across the globe (WHO, 2017), with over 300 million people estimated to suffer from the disease (WHO, 2017). It is characterised by pervasive low mood, loss of pleasure, sleep disturbance and reduced libido, amongst other symptoms (see ICD-10; WHO, 1992). These symptoms of depression impair daily functioning, which can impact on employee retention, and quality of life (Kessler, 2012). The effects of depression can be fatal, with as many as 800,000 people globally dying by suicide (WHO, 2017).

It is apparent from both the economic and personal costs of depression that effective evidence-based interventions are of paramount importance. The recommended treatment for moderate depression is psychological therapy with medication (NICE, 2009). However, the long-term effectiveness of these treatments is subject to debate (Ali et al., 2017; Cuijpers et al., 2013; Knekt et al., 2008). More recently the National Institute for Health and Care Excellence (NICE, 2015) in the UK, and the Food and Drug Administration (FDA, 2008) in the USA, approved the use of repetitive transcranial magnetic stimulation (rTMS) to treat depression. TMS has been repeatedly shown to affect cortical excitability (Huang et al., 2011). However, the precise neural mechanisms of action with respect to depression are unclear (Aleman, 2013; Janicak & Dokucu, 2015; Wassermann & Zimmermann, 2012). There are also major and important questions around its efficacy as a treatment (Chervyakov et al., 2015; Janicak & Dokucu, 2015; Martin et al., 2003; Matheson et al., 2016; Wassermann & Zimmermann, 2012).

The dorsolateral prefrontal cortex (DLPFC) is the region most commonly targeted in the treatment of depression (Lefaucheur et al., 2014), where TMS appears to reduce symptoms of depression (Conelea et al., 2017; Fitzgerald et al., 2009). In particular, a reduction in negative mood is observed, as measured on the Hamilton depression scale (Chen et al., 2013). However, improvement of symptoms is inconsistent across studies and individuals (Fox et al., 2013; see review by Loo & Mitchell, 2005). There is also little consensus amongst studies regarding the most effective frontal region to target: the left, bilateral, or right prefrontal cortex (Chen et al., 2017; Li et al., 2014). The evidence base also shows considerable variation for the number of treatment sessions provided (George et al., 2009) and the type of TMS protocols administered (e.g., Bakker et al., 2015; Chung et al., 2015; Duprat et al., 2016b, Duprat et al., 2016a). Further discrepancies between treatment protocols extend to the frequencies of the rTMS applied, i.e. low-frequency TMS (i.e. <1 Hz; Liu, Zhang, Zhang, & Li., 2014) versus higher-frequency TMS (Fitzgerald et al., 2009; Liu et al., 2014). Additionally, multi-frequency protocols such as Theta Burst Stimulation are increasingly being used due to their relative efficiency and shorter duration (Bakker et al., 2015; Bulteau et al., 2017; Chung et al., 2015; Duprat et al., 2017). Predominantly through demonstrations of effects upon motor cortical excitability, low-frequency (LF; ∼ <1 Hz) TMS has been associated with reduced cortical excitability, whilst high-frequency (HF; ∼ >5 Hz) TMS is associated with excitation of neural activity (Huang et al., 2005; Siebner & Rothwell, 2003). However, despite these opposing effects on physiology, a meta-analysis conducted by Chen et al. (2013) indicated both high and low-frequency TMS yielded similar reductions in depressive symptoms. Despite the similar efficacy of LF-TMS and HF-TMS (see RCT by Eche et al., 2012) the majority of the current protocols use HF-TMS over the left DLPFC to increase excitability in the treatment of depression (Allan et al., 2012; Wassermann & Zimmermann, 2012).

More recent rTMS protocols have begun using intermittent Theta Burst Stimulation (iTBS) to stimulate the left DLPFC (Duprat et al., 2016b, Duprat et al., 2016a; Duprat et al., 2017). iTBS combines low and high-frequency stimulation and has been shown to be relatively effective in reducing the TMS intensity required to produce a motor evoked response, indicating increased excitability (Huang et al., 2005). However, the precise frequency range of iTBS protocols used in depression treatment is variable (cf. Blumberger et al., 2018; Bulteau et al., 2017; Duprat et al., 2017; Fitzgerald et al., 2018; Huang et al., 2005). The development of TMS protocols for treating depression is promising, yet the variation between competing treatment protocols has led to inconclusive results. Despite variation between protocols (iTBS, low-frequency rTMS; high-frequency rTMS) all competing treatment protocols are endorsed by NICE (2015) and the FDA (2008).

Common symptoms of depression that have been linked to the functioning of the DLPFC include reduced reward responsiveness and anhedonia (Ballard et al., 2011; Staudinger et al., 2011). In particular, the DLPFC appears to be innervated via dopaminergic pathways (Der-Avakian & Markou, 2012; Fidalgo et al., 2014) and is thought to play a role in external reward anticipation (Ballard et al., 2011). It has been suggested that facilitatory rTMS applied to the left DLPFC stimulates the mesolimbic reward pathway (Janicak & Dokucu, 2015), which is hypoactive in depression (see review by Belujon & Grace, 2017; Koenigs & Grafman, 2009). The probabilistic learning task (PLT; Pizzagalli et al., 2005) has been robustly related to anhedonia (Pizzagalli et al., 2008; Pizzagalli, et al., 2005) and reward processing in healthy and depressed individuals (Huys et al., 2013). It has also been associated with neural areas such as the left DLPFC, which have been linked to reward responsiveness using fMRI (Der-Avakian & Markou, 2012; Ott et al., 2011) and electrophysiological approaches (e.g., Pizzagalli, et al., 2005). More recently the probabilistic learning task has been used to measure reward responsiveness following rTMS stimulation (Ahn et al., 2013; Duprat et al., 2016b, Duprat et al., 2016a; Duprat et al., 2017).

One of the most important pieces of evidence that has contributed to the advancement of rTMS to treat depression is the work of Ahn et al. (2013). The authors applied facilitatory HF-TMS to the left DLPFC in a control population, leading to heightened reward processing following rTMS compared to sham. Duprat et al., 2016b, Duprat et al., 2016a attempted a partial replication of Ahn et al.’s (2013) study, also using the probabilistic learning task, and did not find an increase in reward processing in comparison to an alternative control condition. Nevertheless, reward responsiveness appeared to be modulated by participants' trait hedonic capacity (Duprat et al., 2016b, Duprat et al., 2016a), which was taken as further support for the use of facilitatory TMS as a treatment. If such an effect replicates, it might provide further support for the recent suggestion of a personalised approach in the use of rTMS as a treatment (Singh et al., 2019). However, the inconsistency in the effect between Ahn et al., (2013) and Duprat et al., 2016b, Duprat et al., 2016a, and the post-hoc nature of the relationship to trait capacity, challenges the reliability of this evidence. Despite the inconsistency between these findings, both of these studies have been used to justify the use of TMS on patients as treatment for depression (e.g., Blumberger et al., 2018; Duprat et al., 2017).

Taken together, the above evidence indicates that the ability of TMS to alter neuronal functioning may impact key markers of depression (anhedonia and reward sensitivity), despite the mixed evidence. This promising avenue for treatment comes at a time when both the efficacy and cost of traditional treatments of depression are a concern (Ali et al., 2017; Friborg & Johnsen, 2017; Johnsen & Friborg, 2015). For example, the long-term effectiveness of low intensity Cognitive Behavioural Therapy, once thought to be the gold standard (Layard & Clark, 2014), is now being questioned (Ali et al., 2017). In addition, the efficacy of antidepressant medication compared to placebo for depression is also mixed (cf. Cipriani et al., 2018; Kirsch, 2014). There is a need for novel treatments that show both clinical utility and long-term effectiveness, which TMS may be able to answer.

Here, we examine the utility of TMS as a method to affect reward sensitivity, a key component of depression, aiming to reconcile the discrepant findings in the existing literature (Ahn et al., 2013; Duprat et al., 2016b, Duprat et al., 2016a) by conducting a replication of Ahn et al., (2013). We include additional measures of trait hedonic capacity and apply the increasingly utilised excitatory iTBS from Duprat et al., 2016b, Duprat et al., 2016a, to redress the discrepant findings (described further in the aims and methods section). Additionally, neither study directly measured whether low mood was modulated as a consequence of TMS, as would be expected (e.g., Blumberger et al., 2018; Bulteau et al., 2017; Duprat et al., 2017). This replication attempt is important for two reasons. First, the protocols used in the studies had key differences that could impact the efficacy of TMS as a treatment for depression. Secondly, in light of the current replication crisis in psychological research (Chambers et al., 2015; Nosek et al., 2015), which is compounded in clinical trial research (Driessen et al., 2015), reproducing effects that have clinical implications is essential.

### 1.1 Aims and hypotheses of the current study

Based on the current use of facilitatory TMS to treat depression (Blumberger et al., 2018; Bulteau et al., 2017) and the inconsistent effects between Ahn et al., (2013) and Duprat et al., 2016b, Duprat et al., 2016a replication, we conducted a replication of Ahn et al.’s (2013) study. The study uses the probabilistic learning task and extends it through the inclusion of measures of mood. Any additional deviations from replication are described in the methods and are only applied where there are substantial concerns over the original methods used (e.g., to have a baseline measure of probabilistic learning task prior to and post stimulation Ahn et al., 2013, see methods). As iTBS (Duprat et al., 2016b, Duprat et al., 2016a; Duprat et al., 2017) is starting to be more commonly used in the treatment of depression than the HF-TMS protocol used by Ahn et al., (2013), this replication applies iTBS (Blumberger, 2018; Bulteau et al., 2017; Duprat et al., 2017). Comparisons are made to a sham control.

#### 1.1.1 Primary hypothesis

We predicted an increase in response bias (RB), as a function of active compared to sham stimulation, the basic expression of which would be an elevation in RB in block one following active stimulation compared to the equivalent sham stimulation. This is our critical effect of interest and was demonstrated by Ahn et al., (2013) in the first block of their probabilistic learning task. Our prediction was theoretically grounded in the multiple treatment studies that have found that active stimulation decreases depressive symptomatology, such as anhedonia, compared to sham stimulation (cf. Fitzgerald et al., 2009; George et al., 2009; Lam, Chan, Wilkins-Ho, & Yatham, 2008; O’Reardon et al., 2007).

#### 1.1.2 Secondary hypotheses

We expected scores from the secondary questionnaire, which measures mood (Positive and Negative Affect Scale; PANAS; Watson et al., 1988a, Watson et al., 1988b), to show a reduction in negative mood and increase in positive mood respectively. In particular, we expected active compared to sham TMS will modulate mood ratings, as measured on the PANAS (Watson et al., 1988a, Watson et al., 1988b). In line with previous depression intervention studies measuring mood (e.g., Chaves et al., 2017) and rTMS (Moulier et al., 2016) we expect rTMS to decrease negative affect and increase positive affect (see secondary analysis section for further detail and justification).

#### 1.1.3 Replication interactions of interest

In addition to the above hypotheses, Ahn et al., (2013) and Duprat et al., 2016b, Duprat et al., 2016a demonstrated significant interactions and secondary effects consistent with TMS having a positive effect in reducing symptoms of depression, as measured using RB from the probabilistic learning task. However, neither Ahn et al.’s (2013) nor Duprat et al., 2016b, Duprat et al., 2016a significant interactions are fully in line with the theoretical prediction that active stimulation will increase RB in the probabilistic learning task, compared with sham stimulation. Ahn et al., (2013) reported a significant interaction between block and stimulation in which RB increased for active stimulation in *block 1,* but interestingly also increased following *sham* stimulation compared to *active* for *block 2* of the task. Similarly, Duprat et al., 2016b, Duprat et al., 2016a main significant interaction of time (pre/post stimulation) × stimulation (active/sham) × block (1,2,3) was only apparent when, TEPS-CON measuring hedonic capacity, was added as covariate (see Methods; Questionnaires). This indicated an increase in RB as a pre-stimulation baseline for active compared to sham stimulation when hedonic capacity was taken into account. For completeness of this replication, we test for the exact replication of these secondary interactions.

## 2 Method

### 2.1 Task overview & procedure

**Fig. 1:**
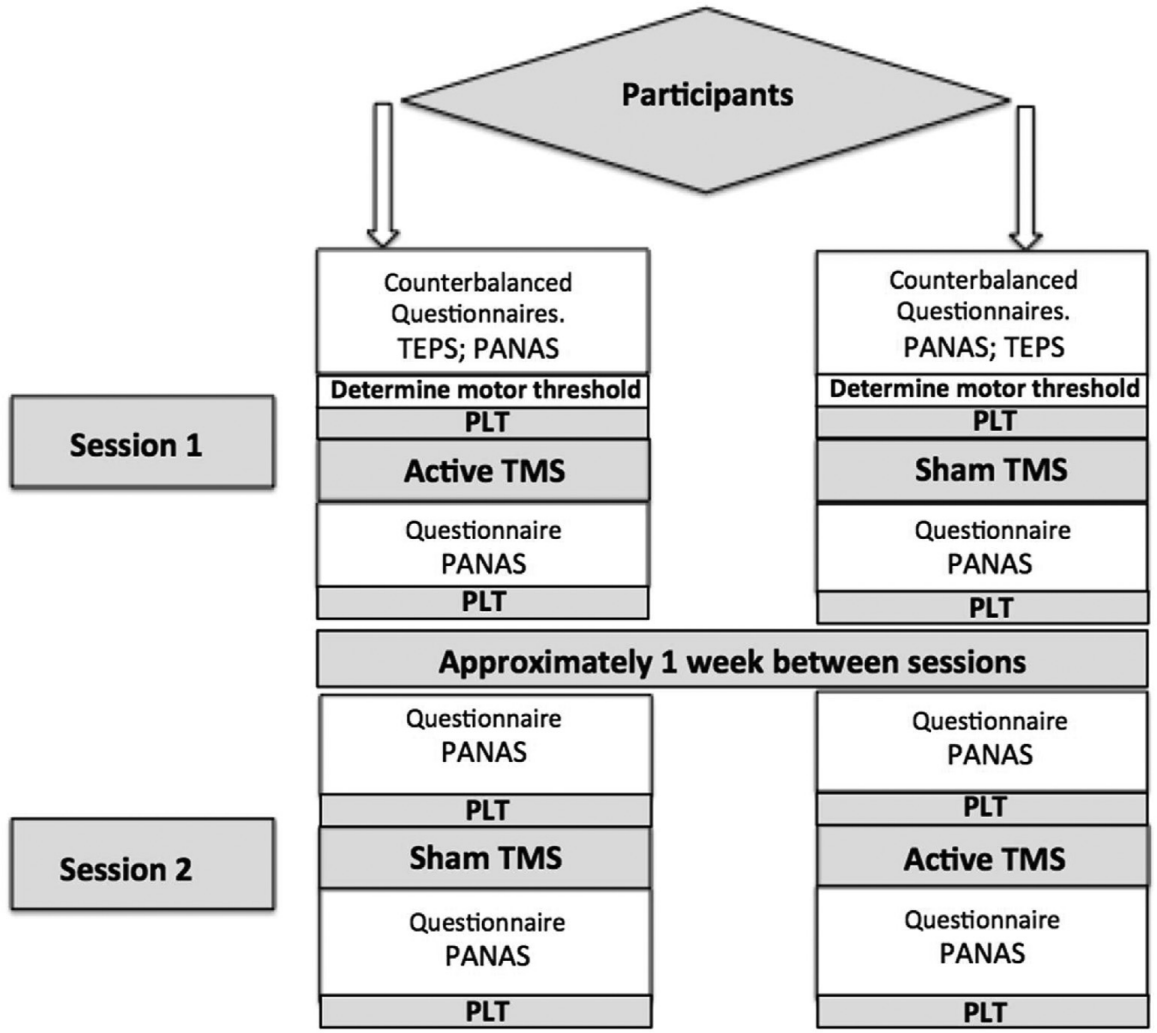
Task procedure for participants entering the study. TEPS = Temporal Experience Pleasure Scale questionnaire; PANAS = Positive and Negative Affect Scale; PLT = Probabilistic Learning Task; TMS = Transcranial Magnetic Stimulation.

**Fig. 2:**
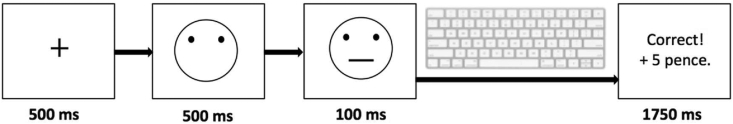
Trial schematic in the probabilistic learning task (based on Pizzagalli et al., 2005).

The iTBS protocol used by Duprat et al., 2016b, Duprat et al., 2016a; 2017) was used to stimulate the DLPFC (for more detailed explanation see ‘Transcranial Magnetic Stimulation’ section, below). Participants were asked to attend two testing sessions (see Fig. 1 for precise procedure), and were assigned to either a sham or active iTBS condition, determined through initially flipping a coin (see Duprat et al., 2016b, Duprat et al., 2016a). The respective sides of the coin were assigned “sham” or “active” (e.g., heads = sham; tails = active). If participants were assigned to the active condition during the first session, they received sham stimulation during the second session, and vice versa. To determine participants' reward learning, participants completed a PLT (Pizzagalli et al., 2005) prior to iTBS stimulation. Participants were asked to complete a further PLT following TMS stimulation (see Fig. 2).

**Fig. 3:**
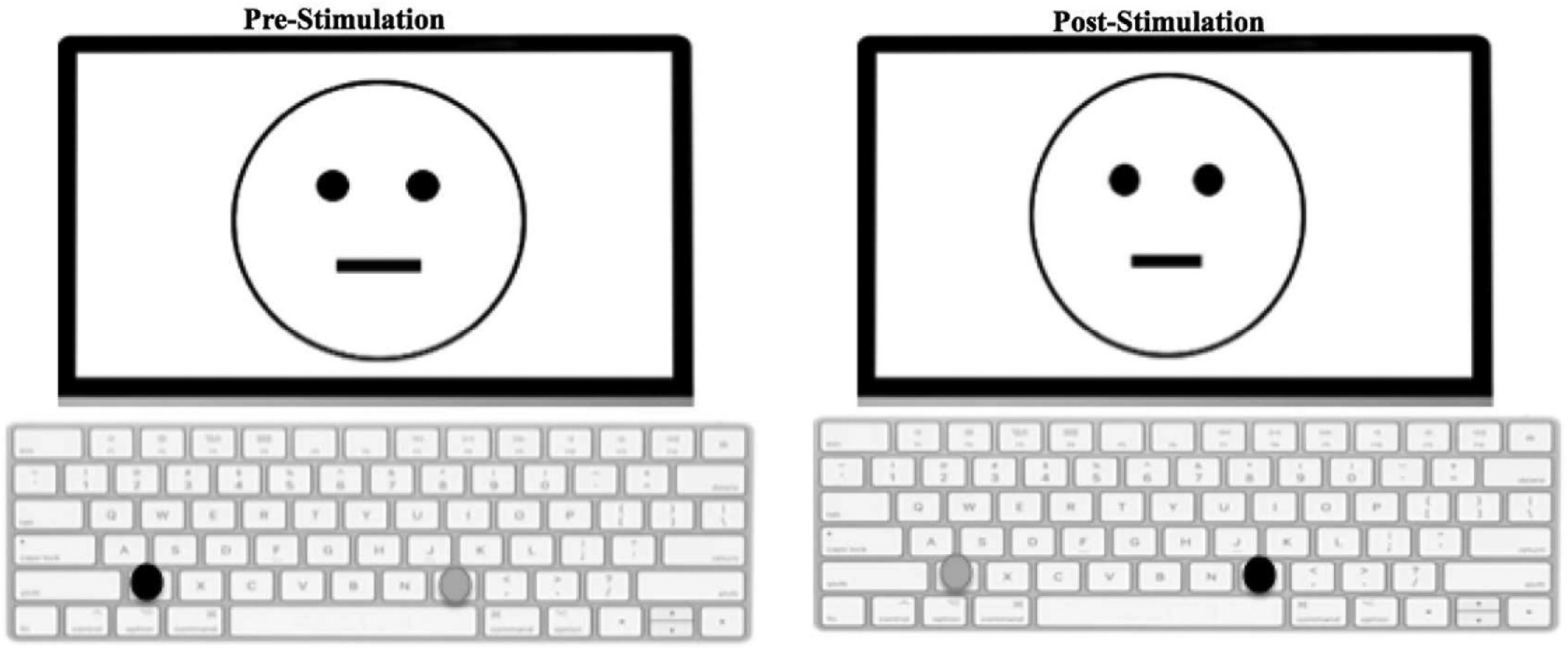
Counterbalancing procedure for sessions: pre and post stimulation. Keyboard presses “Z” and “M” were counterbalanced within a testing session and between participants. The main experimental manipulation of “rich” and “lean” stimuli was counterbalanced within stimulation sessions. The pairing was determined randomly at the start of the stimulation session and counterbalanced across each stimulation session.

During the first session, participants were asked to complete the Temporal Experience of Pleasure Scale questionnaire (TEPS; Gard, Gard, Kring, & John, 2006) prior to the TMS stimulation, and the PLT task. In both sessions participants were asked to complete a further questionnaire, to determine whether mood ratings have changed before and after TMS stimulation (PANAS; Watson et al., 1988a, Watson et al., 1988b; see ‘Questionnaire section’ for more detailed information). The PANAS was undertaken both before the PLT, before stimulation, and again before the PLT following stimulation.^1^ This is a deviation from the procedures of Ahn et al., (2013) and Duprat et al., 2016b, Duprat et al., 2016a, however, applying the PANAS before and after stimulation^2^ avoided the potential concern that the PLT might affect the PANAS mood ratings.

Participants were paid £10 per hour, and a maximum of £12 on the reward-learning tasks. The ethics committee at Cardiff University's School of Psychology has approved the study.

### 2.2 Study design

The study is a repeated measures design, with participants undergoing all conditions; 2 (Stimulation: Active and Sham), 2 (Time: Pre and Post stimulation), 2 (Condition: Rich and Lean, see section 2.2.1.) and 3 (Block: One, Two, Three). In accordance with Duprat et al., 2016b, Duprat et al., 2016a, we administered the hedonic capacity questionnaire, TEPS (Gard et al., 2006) (see Primary hypothesis 1.1.1).

We also examined the effect of rTMS (sham, active) on mood. Ratings from the PANAS (Watson et al., 1988a, Watson et al., 1988b) were collected before and after stimulation (see secondary hypotheses, see the Secondary Analyses section).

#### 2.2.1 Probabilistic learning task

The PLT is based on signal detection theory (McCarthy & Davison, 1979) and measures an individual's decision to choose stimuli A over stimuli B (Pizzagalli et al., 2008b, Pizzagalli et al., 2008c; Pizzagalli et al., 2005) based on a prior reinforcement learning schedule. Participants receive rewards that vary based on an asymmetric reinforcement schedule (e.g., ‘rich’ or ‘lean’ stimuli; Pizzagalli et al., 2005). Previous literature has suggested that reward learning is biased towards the most rewarded stimuli (Pizzagalli et al., 2008; Pizzagalli et al., 2005). Individuals with depression tend to display a lower RB to more frequently rewarded stimuli compared to non-depressed controls, suggesting difficulty with reinforcement leaning (Pizzagalli et al., 2008a, Pizzagalli et al., 2008b, Pizzagalli et al., 2008c).

Each application of the task (four in total, two in session one, and two in session two; see Fig. 1) comprised of three blocks of 100 trials (Block 1, Block 2, Block 3). For each trial, a fixation cross appeared on the screen for 500 ms, followed by a cartoon face without a mouth for 500 ms. Another cartoon face was subsequently presented on the screen with either a ‘long’ (13 mm) or ‘short’ (11.5 mm) mouth for 100 ms. The participant provided a keyboard response to assign whether the mouth was ‘long’ or short’. If correct, a feedback screen was presented for 1750 ms, which was either blank or announced that the participant had won five pence. For each block of 100 trials, a pseudo random sequence of 50 long and 50 short mouths was presented.

One mouth type, the “rich” condition, was selected at random to be rewarded three times as often as the other type of mouth, the “lean” condition. In total 40 trials were rewarded per block, 30 of these were the “rich” condition and 10 of these were the “lean” condition. The counterbalancing procedure is described in detail below. Participants were asked to try and win as much money as they can. Also, participants were told that not all trials will be rewarded, but they were not told about the rich versus lean stimulus.

The PLT was implemented in PsychoPy (Peirce 2007), and was presented on a Microsoft PC with an Asus LCD monitor (60 Hz refresh rate).

#### 2.2.2 Counterbalancing

For the PLT, several parameters were counterbalanced. To reduce order effects and to control for a laterality bias, the response keys (“Z” or “M”) that participants press were counterbalanced between stimulation sessions. This was conducted through the assignment of odd and even numbered participants receiving opposite keyboard presses. Each of the response keys were paired with a face with a “long” or “short” mouth, which relates to the experimental manipulation of “rich” versus “lean” reinforcement schedules. For the odd numbered participants, the “Z” key was paired with a “long” mouth and the “M” key with a “short” mouth pre-stimulation and the reverse pairing post stimulation (“Z” with “short” and “M” with “long”). The even participants received the opposite pairings. Within each stimulation session, the face that was rewarded richly or leanly–short or long was chosen at random (using Python's *shuffle* function) at each pre-stimulation session. Then, at post-stimulation sessions, the ordering was reversed. Participants were also asked to press “Z” key with their left index finger and the “M” key with their right index finger (see Fig. 3).

### 2.3 Transcranial magnetic stimulation

Following the iTBS protocol of Duprat et al., 2016b, Duprat et al., 2016a, we applied iTBS stimulation using a Magstim Rapid^2^ stimulator (Magstim Company Limited, Wales, UK), which was connected to a 70 mm “figure eight” shaped cooled coil (P/N 3910–00). We used the Brainsight neuronavigation system (Brainsight Rogue Research, Inc.) to accurately target the left DLFPC. In the instances of MRI scans not being available, we used the default MNI average brain scan in Brainsight 2.3, (Rogue Research, Inc.) to target the left DLPFC. The study that is the target of our replication, Ahn et al. (2013), used a standard location for the DLPFC for each participant, based on anatomical landmarks and position relative to motor areas.^3^ We aimed to use participants' MRI scans, where available, to take into account individual structural differences between participants. The target for the DLPFC was identified in Montreal Neurological Institute (MNI) coordinates as *x* = −27, *y* = 30, *z* = 38, of which the mean of DPLFC co-ordinates were reported as being active in a perceptual decision-making task (Heekeren et al., 2004), and targeted in a TMS based cognitive control intervention (Hayashi et al., 2013). As in Duprat et al., 2016b, Duprat et al., 2016a protocol, individual motor threshold was determined using surface electromyography to produce a motor evoked potential in the right abductor pollicis brevis muscle, during the first testing session.

Participants were asked to take part in two testing sessions, where they received either active or sham iTBS with an interval of approximately one week between the sessions. The active iTBS consisted of: 1620 pulses in 54 cycles of 10 bursts of 3 pulses with a train duration of 2s and inter-train interval of 8s with a power output of 110% of the resting motor threshold.

For the sham stimulation, a sham coil was used (P/N 3950–00). The sham coil was manufactured to have the same visual appearance and auditory artefact as the active coil but did not deliver appreciable magnetic stimulation. Therefore, sham stimulation followed the same procedure as the active condition. Order of stimulation was counterbalanced between participants and initial allocation to active and sham conditions was randomised using a coin toss.

### 2.4 Deviations from Ahn et al., (2013)

Table 1 below highlights the principle deviations from Ahn et al.,’s (2013) protocol.^4^ We describe each major deviation from Ahn et al., (2013) and justify alternative methods e.g. the use of an iTBS protocol, as opposed to the HF-TMS protocol used by Ahn et al., (2013). Note that iTBS is thought to be more efficient than HF-TMS, involving fewer pulses for comparable effects and both the HF-TMS and iTBS protocols, applied respectively in Ahn et al., (2013) and Duprat et al., 2016b, Duprat et al., 2016a, are acknowledged as excitatory TMS protocols (Huang et al., 2011). As the more recently developed iTBS is more efficient, its use in treatment appears to be growing at a faster rate than HF-rTMS and was, therefore, the focus of this replication (Bakker et al., 2015; Grossheinrich et al., 2009).

**Table 1:** Major Deviations from Ahn et al. (2013) protocol, and justification of alternative methods.

Study Element	Ahn et al., (2013)	Duprat et al., 2016b, Duprat et al., 2016a	Current study	Justification
TMS protocol	HFTMS	iTBS	iTBS in accordance with Duprat et al., 2016b, Duprat et al., 2016a	In more recent treatment protocols for depression, iTBS is being used as a treatment (e.g., see Bulteau et al., 2017)
TMS Stimulator	Magstim 200 Magnetic Stimulator	Magstim Rapid^2^ Plus1	Magstim Rapid^2^	Although a different stimulator will be used, with the potential of reduced frequency due to capacitance of the system, the frequencies are well within the range (i.e., ∼10 Hz) to generate excitatory neural changes (e.g., Huang et al., 2005; Siebner & Rothwell 2003).
Number of Probabilistic Learning Tasks	2 (post active and post sham stimulation).	4 (pre and post active and pre and post sham stimulation).	4 probabilistic learning tasks in accordance with Duprat et al., 2016b, Duprat et al., 2016a.	A baseline measure of reward bias allows for the exclusion of the possibility that day-to-day variance might explain observations.
Hedonic Capacity Questionnaire	No measures used to assess hedonic capacity.	Temporal Experience of Pleasure Scale (Gard et al., 2006).	Temporal Experience of Pleasure Scale (Gard et al., 2006).	Based on Duprat et al., 2016b, Duprat et al., 2016a's finding that hedonic capacity interacts with reward responsiveness.
Mood questionnaire	No measures used to assess mood.	No measures used to assess mood.	Positive and Negative Affect Schedule (Watson et al., 1988a, Watson et al., 1988b).	To ensure effects of active compared to sham TMS is related to depressive symptoms, e.g., low mood. See secondary hypotheses section for precise hypotheses.

We also used a Magstim Rapid^2^ stimulator as opposed to a Magstim Rapid^2^ Plus1 used by Duprat et al., 2016b, Duprat et al., 2016a, and Magstim 200 used by Ahn et al., (2013) (see Table 1 and Supplementary information https://osf.io/sep4g). The Rapid^2^ stimulator is the most popular repetitive TMS stimulator sold by Magstim. iTBS comprises of 0.1 Hz, 5 Hz, and 50 Hz components. However, over the course of the experiment it became apparent that this stimulator builds in a small frequency reduction at intensities greater than 50% of the maximum stimulator output. The reduction involves a slight reduction of the 50 Hz component at a rate of -1 Hz per 2% greater than 50% of maximum. This resulted in the obtained mean frequency of the prescribed 50 Hz component being, *M* = 45.967 Hz, *SD* = 3.690 (full details are available is supplementary information at https://osf.io/m2fsg/). As these frequencies are all well above the putative cut of between excitatory and inhibitory frequencies (5 Hz, Huang et al., 2005) and the 10 Hz used by Ahn et al., (2013), we think it unlikely that the reduction could have made a substantive or theoretical difference.

### 2.5 Questionnaires

#### 2.5.1 Temporal Experience of Pleasure Scale (TEPS)

The TEPS consists of 18 self-report items, of which there are two subscales: anticipatory (TEPS-ANT; ten items) and consummatory (TEPS-CON; eight items) pleasure. Consummatory pleasure has been linked to immersive pleasurable experiences (Gard et al., 2006; Gard, et al., 2007), and anticipatory pleasure has been related to reward responsiveness (Gard et al., 2006). The sum of these two scales provides a measure of hedonic capacity. The higher the total score on these scales, the greater the hedonic capacity. Conversely, the lower the total score, the lower the hedonic capacity (anhedonia, e.g. Gard et al., 2007; Strauss et al., 2011). Internal consistency for each subscale has good reliability, consummatory (*α* = .71) and anticipatory (α = .74) (Gard et al., 2006). The TEPS scale has been used with both non-clinical (the target of this replication; Ahn et al., 2013; Duprat et al., 2016a, Duprat et al., 2016b) and clinical populations (e.g., Gard et al., 2006, 2007; Sherdell et al., 2012).

#### 2.5.2 Positive and egative affect schedule (PANAS)

To determine participants' current mood, we used the PANAS (Watson et al., 1988a, Watson et al., 1988b). This scale consists of ten positive (e.g., ‘proud’) and ten negative (e.g., ‘jittery’) adjectives. Participants will rate on a Likert scale (1–5), the extent to which they currently feel that emotion. Scores on the PANAS range between 10 and 50, on both positive and negative subscales separately. A relatively higher score on the positive affect scale (PA) reflects a more positive mood, whereas a higher score on the negative affect (NA) subscale reflects a more negative mood (see Watson et al., 1988a, Watson et al., 1988b). Each subscale of the PANAS has good internal reliability, PA (*α* = .89) and NA (*α* = .85) (Crawford & Henry, 2004). The PANAS has been used with both clinical populations (people diagnosed with depression and anxiety) and in non-clinical populations (Watson et al., 1988a, Watson et al., 1988b). Lower scores on the PA scale have been related to anhedonia and depression (e.g., Crawford & Henry, 2004; Watson et al., 1988a, Watson et al., 1988b). Higher scores on the NA scale have been related to depression and anxiety disorders (e.g., Crawford & Henry, 2004). The PANAS has good discriminant and convergent validity (Crawford & Henry, 2004).

### 2.6 Primary dependent variable–probabilistic learning task: response bias

The RB relates to the participant's preference to the most frequently rewarded stimulus (“rich”) when compared to the least rewarded stimulus (“lean”). RB was calculated using the formula below. The response rate will increase if the participant selects “rich” stimuli more frequently than “lean” stimuli, regardless of accuracy. In accordance with previous studies, i.e. Ahn et al. (2013), Duprat et al., 2016a, Duprat et al., 2016b, Pizzagalli et al. (2005), RB is likely to increase between blocks 1 & 2 as a consequence of reinforcement learning. The number of trials presented in the PLT are equal to those used in previous studies applying the PLT (e.g., Ahn et al., 2013; Chevallier et al., 2016; Duprat et al., 2016b, Duprat et al., 2016a; Lancaster et al., 2012; Lancaster et al., 2015; Pizzagalli et al., 2008; Pizzagalli et al., 2005). All analyses will be administered according to Pizzagalli et al., (2005), Ahn et al., (2013), and Duprat et al., 2016b, Duprat et al., 2016a manuscripts. Similar to Ahn et al., (2013), effect sizes were reported using partial eta-squared (*η*_*p*_^2^). In accordance with Duprat et al., 2016b, Duprat et al., 2016a, we also calculated Cohen's *d* to evaluate effect sizes. In line with Duprat et al., 2016b, Duprat et al., 2016a, if the assumption of sphericity was violated Greenhouse Geiser correction was applied to the data.

$$RB=\left[\log\;b=\frac{1}{2}\log\left(\frac{Rich\;correct\;*\;Lean\;incorrect}{Rich\;incorrect\;*\;Lean\;correct}\right)\right]$$

### 2.7 Analyses

#### 2.7.1 Main analyses: primary hypothesis

We designed our replication attempt to find an effect that uses the same task (the PLT) and stimulation (active, sham) as the target publications (Ahn et al., 2013; Duprat et al., 2016b, Duprat et al., 2016a), and is consistent with the evidence-base that RB will increase as a function of active TMS. Therefore, our main critical one-degree of freedom test of interest is based on that of Ahn et al.’s (2013) direct comparison of the effects of active compared to sham stimulation on RB for Block 1 of the PLT (active block1 – sham block 1). As our primary analysis, we calculated a Bayes Factor applied to our data modelling H1 as a *t*-distribution using the mean difference and *SE* equivalent to those observed by Ahn et al., (2013) for their corresponding one degree-of-freedom (1df) test of interest (*M* = .14; *SE* = .063, *df* = 17) (Dienes & Mclatchie, 2018). In addition, we computed a complimentary frequentist paired *t*-test for active compared to sham stimulation for RB in Block 1. The Bayesian statistic was the primary decision statistic reported.

#### 2.7.2 Secondary analyses: mood questionnaire

Our secondary hypothesis that active compared to sham TMS will increase positive mood and decrease negative mood was based on Chaves et al.’s (2017) clinical trial study. Amongst other variables, Chaves et al., (2017) measured mood using the PA and NA subscales of the PANAS questionnaire, before and after psychological therapies such as Cognitive Behaviour Therapy (CBT).

For our secondary analyses, to assess the evidence that active stimulation compared to sham will (a) increase positive affect, and (b) decrease negative affect, we calculated Bayes Factors (Dienes & Mclatchie, 2018) modelling H1 *t*-distribution as the mean difference and *SE* observed by Chaves et al., (2017) for treatment effects on (a) changes in positive affect (*M* = 4.91, *SE* = .82, *df* = 95) and (b) changes in negative affect (*M* = −5.92, *SE* = .94, *df* = 95) (Dienes, 2008; Dienes & Mclatchie, 2018). To obtain a value that could be used in a 1df test, we subtracted the post PANAS PA score from the pre PANAS PA stimulation ratings for both active and sham stimulation separately, and subsequently subtracted stimulation type (active–sham). More positive scores for this index relate to higher positive mood. PANAS NA values were computed similarly. More positive scores on the PANAS NA index indicate negative mood had decreased. We also conducted two paired sample *t*-tests as complimentary frequentist statistics comparing post active and sham stimulation, one applied to the positive affect measure and the other applied to the negative affect measure.^5^

#### 2.7.3 Replication interactions of interest

The previous critical effects of interest in both Ahn et al., (2013) and Duprat et al., 2016b, Duprat et al., 2016a were significant interactions. Ahn et al., (2013) reported a significant 2 × 3 interaction between Stimulation (active, sham) and Block (1,2,3) for reward learning. Duprat et al., 2016b, Duprat et al., 2016a reported a significant 2 × 2 × 3 interaction (with the presence of the TEPS-CON covariate) for Time (pre, post) × Stimulation (active, sham) × Block (1,2,3 on the reward learning task). While these are consistent with the primary outcome of active TMS increasing reward responsiveness compared to sham (main analyses), these interactions are not the basic effects one would expect of active TMS having an effect on the PLT. Therefore, the interactions reported by Ahn et al., (2013) and Duprat et al., 2016b, Duprat et al., 2016a are of secondary interest.

To test for the interactions described by Ahn et al., (2013) and Duprat et al., 2016b, Duprat et al., 2016a, we computed a Bayes Factor for both of their reported interactions. Following the method outlined in Dienes (2014) for reducing interaction effects to a one-degree of freedom test, for Ahn et al.’s (2013) primary finding of a Stimulation × Block interaction, we computed the differences between active and sham in each block, then subjected these scores to a linear contrast (e.g., B1 + −.5×B2 + −.5×B3) to reduce the interaction term to a one-degree of freedom test. We subsequently used the Bayes Factor calculator (Dienes & Mclatchie, 2018) to apply the parameters calculated from Ahn et al.’s (2013) primary interaction: *M*_*diff*_ = .16, *SE* = .08 and the obtained *M*_*diff*_ and *SE* for the interaction from our study, reduced to a one-degree of freedom test as described above.

For Duprat et al., 2016b, Duprat et al., 2016a interaction (Time × Stimulation × Block with the added covariate of hedonic capacity; TEPS-CON), we used the same procedure as we described above to reduce the significant interaction into its constituent one-degree of freedom test, prior to using these parameters in the Bayes Factor calculator (Dienes & Mclatchie, 2018). We computed the linear contrast between active and sham in each block, once for each level of the ‘Time’ factor, and then computed the differences between these two scores, reducing the comparison to a simple difference score. To account for the presence of the covariate, we used the covariate-adjusted means (as were reported for Duprat et al., 2016b, Duprat et al., 2016a's interaction term above), to calculate our one-degree of freedom values. That is, we computed a repeated-measures ANCOVA with the factors (Time × Stimulation × Block with the covariate) to obtain the resulting adjusted means to compute our mean differences (similar to Dienes, 2014), and SE for calculating the resulting Bayes Factor. These were integrated with a prior based on the results obtained from reducing Duprat et al., 2016b, Duprat et al., 2016a results into a one-degree of freedom test; *M*_*diff*_ = .14, SE = .07 We will also report all frequentist equivalents of these statistics, as well as a full exposition of the ANOVAs and interactions.

#### 2.7.4 Manipulation checks

We conducted an outcome neutral test, where we compared participants’ RB scores from block three of the PLT to the value of zero, using a one-sample *t*-test. At the group level, this was achieved by computing the average RB in block three for the post sham stimulation session, before submitting the scores to a one-sample *t*-test. This test will reveal whether participants have a RB significantly larger than zero, indicating if they are responding more to rich compared to lean stimuli, after undergoing the first and second blocks of the study. Taking the values from block 3 following sham stimulation will provide a baseline measure of the propensity to acquire a RB. We also computed the Bayesian equivalent of this one-degree of freedom test, that is RB for block 3 of the PLT compared to the numerical value of zero, applying priors based on the mean of the effects and standard errors reported for block three of post sham stimulation for both Ahn et al., (2013) (*M* = .19, *SE* = .061, *SD* = .26, *df* = 17) and Duprat et al., 2016b, Duprat et al., 2016a (*M* = .26, *SE* = .070, *SD* = .32, *df* = 20) respectively.

### 2.8 Bayes Factors and sample size estimates

#### 2.8.1 Primary hypothesis

We used a Bayesian approach (Dienes, 2014) to estimate the likely sample size needed to provide support for the null or alternative hypothesis, related to our critical effect of interest. Assuming a difference of zero and the same *SE* and sample size reported in Ahn et al., (2013), and applying the *R* Code (Dienes & Belfi, 2019) we were able to estimate the Bayes Factor for our proposed sample size of 30 under the null as .14. This was computed through scaling the *SE* (.063) in Ahn et al., (2013) by the square root of their sample size divided by our proposed sample size (*SE*_scaled_ = .049) for use in the Bayes Factor function. As we are closely replicating Ahn et al., (2013) study, we have made the assumption that our testing procedures will be similar and should, therefore, yield no additional variance.

#### 2.8.2 Secondary analysis

For our secondary analysis that active compared to sham stimulation will increase positive affect and decrease negative affect, using the *R* Code (Dienes & Belfi, 2019), and assuming no effect and the *SE*, and sample size reported in Chaves et al., (2017), we were able to estimate the Bayes Factor for our proposed sample size of 30 under the null as .01 for negative affect, measured on the PANAS-NA; and the Bayes Factor of .02 for positive affect, as measured on the PANAS-PA.

#### 2.8.3 Previous critical interactions of interest

We computed the Bayes Factor for the significant interactions reported in Ahn et al. (2013) and Duprat et al., 2016a, Duprat et al., 2016b following the method outlined in Dienes (2014) and detailed further in our main analyses section above*.* Using the *R* Code (Dienes & Belfi, 2019), sample size, and *SE*s calculated for Ahn et al., (2013) interaction (Stimulation × Block) we estimated a Bayes factor of .17 under the null for our sample size of 30. The Bayes Factor was computed through scaling the *SE* (.08) for Ahn et al.’s (2013) interaction by the square root of their sample size divided by our sample size (*SE*_scaled_ = .06). We followed the same procedure for Duprat et al., 2016b, Duprat et al., 2016a significant interaction (described further in our analysis section). For Duprat et al., 2016b, Duprat et al., 2016a, the Bayes Factor we obtained under the null was .21, using their *SE* (.07; with a *df* = 20), scaling to our sample size of 30 (*SE*_scaled_ = .06).

#### 2.8.4 Manipulation checks

Using the method described above in the corresponding Analysis section, and *R* code produced by Dienes and Belfi (2019) we estimated a Bayes Factor of .04 under the null for our sample size of 30, using the sample size, *SE* and *df* for the RB reported in block 3 for Ahn et al., (2013) post sham stimulation (*M* = .19, *SD* = .26, *SE* = .061). Similarly for Duprat et al., 2016b, Duprat et al., 2016a, we used the reported mean for RB in block 3 post sham stimulation (*M* = .26, *SD* = .32, *SE* = .070) and obtained a Bayes Factor of .02 under the null after scaling the SEs of the sample to our maximum sample size of 30.

Given the Bayesian approach adopted we continued collecting data until the resultant Bayes factors for primary and secondary analyses were all greater than 6 or less than 1/6 or we had collected data from 30 participants, due to feasibility constraints and in line with the above calculations. Bayes factors greater than 6 were interpreted as substantial evidence for the hypotheses and Bayes factors less than 1/6 were interpreted as substantial evidence for the null (Dienes, 2011; Jeffreys, 1998). This cut off aligns with *Cortex's* guidelines.

### 2.9 Exclusion criteria

If participants did not pass initial safety measures pertaining to TMS approved by Cardiff University, they did not participate in the experiment (Allen et al., 2018; Maizey et al., 2013). In line with Duprat et al., 2016b, Duprat et al., 2016a, should individuals not complete the task appropriately (e.g., only pressing one key throughout the experiment) they were not included in any analyses. In addition, should participants' reaction time be too quick (i.e., under 200 ms) or too slow (i.e., over 2000 ms) those responses were not included in any analyses (Ahn et al., 2013), if this occurred on greater than 10% of trials the participant's data was excluded. Participants were free to withdraw for any reason. Unanticipated technical failings could also result in participant data being excluded. If a participant only completed one testing session, their data was not included in the analyses. We tested further participants to replace any excluded.

## 3 Results

Data, materials, and pre-registration protocol are available at https://osf.io/724gt/.

### 3.1 Participants

Forty-one participants were recruited from a TMS participant database at Cardiff University Brain Research Imaging Centre (CUBRIC) at Cardiff University. All participants had undergone safety screening for contra-indications of TMS. Thirty participants (M*age* = 23.333; SD = 4.198; 20 Females/10 Males) were included in the final TMS analyses, with eleven excluded. Of the eleven excluded, five were excluded for technical reasons–TMS internal cooling mechanism was not operating correctly and the coil overheated, which was then repaired by the manufacturer and did not affect the subsequent sessions. When overheating occurred, the iTBS did not run to completion. As exposure to part of the iTBS protocol, or the learning within the task, could have been affected by repeating the session, the affected participants were excluded. Six participants withdrew voluntarily as they reported discomfort on exposure to active iTBS protocol and did not wish to continue. All participants were right-handed, spoke English fluently and proficiently and had no history of mental health difficulties. The assignment to stimulation ordering (active then sham and the converse) was evenly split across the sample, with 15 participants in each group.

### 3.2 Primary hypothesis

**Fig. 4:**
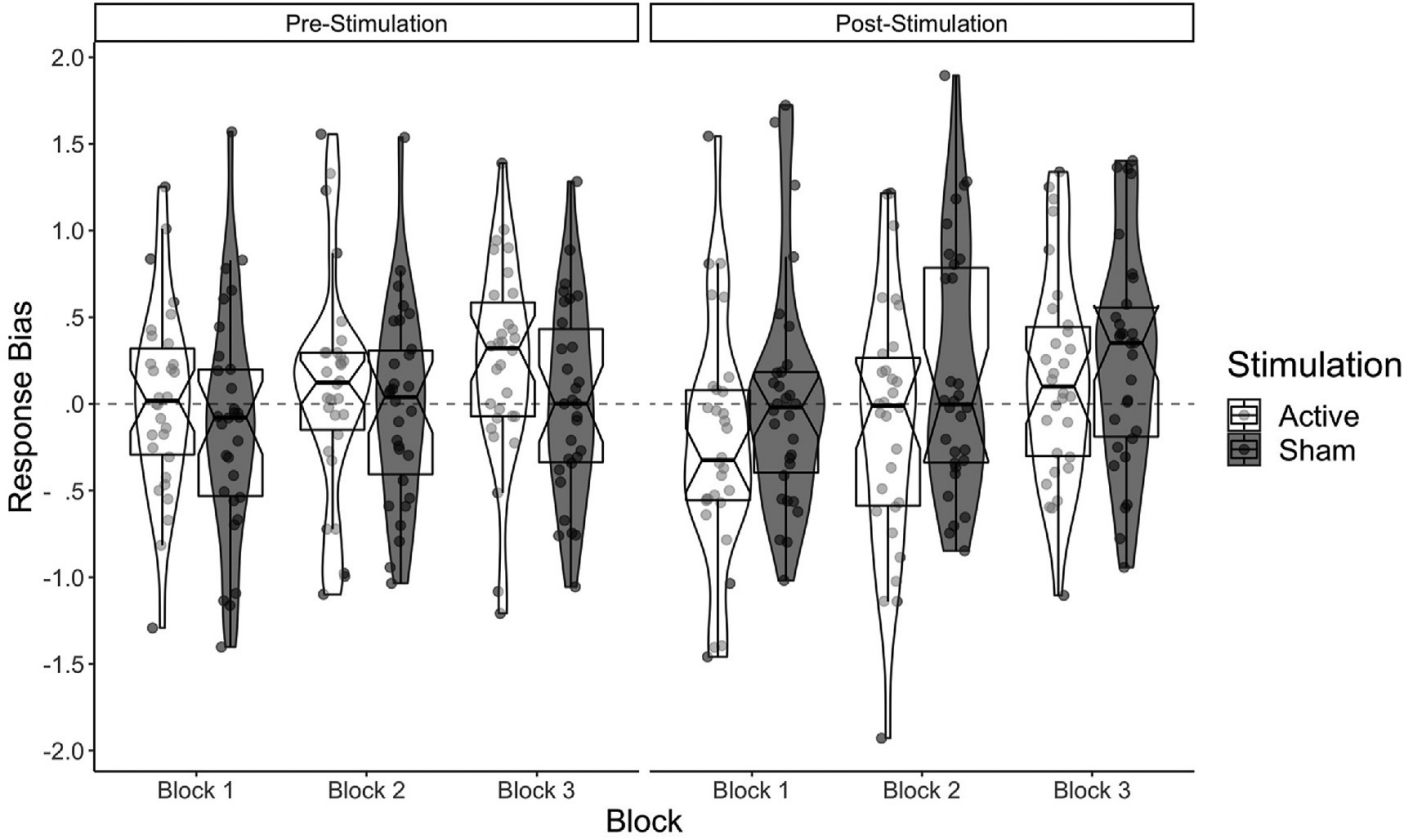
Graph depicting the raw data and distributions of response bias across all conditions (time: pre, post; block: 1,2,3; stimulation: active, sham).

The critical test was a direct comparison of the effects of active and sham stimulation on the RB measure for Block 1 of the PLT (active block1 – sham block 1, see also Fig. 4). We computed a Bayes Factor applied to our observed data (*M* = −.253, *SE* = .187) with a two-tailed prior based on the mean difference and *SE* observed by Ahn et al., (2013) for their corresponding one *df* test of interest (*M* = .14; *SE* = .063, *df* = 17, *SD* = .26). With a group mean in the opposite direction to that predicted under the replication, the resultant BF was = .696, which indicates an insensitivity in terms of a strong conclusion, but what evidence there is favours the null. Our complimentary frequentist paired-sample *t*-test indicated a non-significant difference between RB for block 1 of the PLT for post active (*M* = −.232, *SE* = .124) compared to post sham (*M* = .021, *SE* = .121) stimulation, *t*_(29)_ = −1.353, *p* = .186, *d* = −.247. These results indicate no conclusive support for either the alternative or null hypothesis.

#### 3.2.1 Exploratory analysis

To explore the primary analysis we also applied a directional one-tailed Bayesian 1df test, with the prior modeling an increase in response bias following active TMS, resulting in a BF = .217, indicating an absence of effects in this direction.

Following data collection, we, and a reviewer, noticed that the mean for response bias was below zero in the post active condition in block one (*M* = −.232, *SE* = .124). This raised the possibility that the TMS could have contributed to learning the rewarded stimulus of the pre-TMS block, leading to a drop in bias following stimulation when the rewarded pairing was changed. However, the evidence for such a drop relative to zero was inconclusive, BF = 1.11 (evidence for the null hypothesis, from an exploratory one-sampled Bayesian *t*-test using a default Cauchy prior with scale set at .707), and non-significant, *t*_(29)_ = 1.87, *p* = .07. Furthermore, Duprat et al., 2016b, Duprat et al., 2016a noted a similar drop, but in their post sham condition, collectively suggesting large sample to sample variability with such measures.

### 3.3 Secondary hypothesis

#### 3.3.1 Positive affect

The BF representing evidence for increased positive affect relative to the null, with a prior based on Ahn et al.’s (2013) report, was .222 (*M* = −1.333 and *SE* of our sample = 1.016), indicating stimulation did not modulate positive mood.

**Fig. 5:**
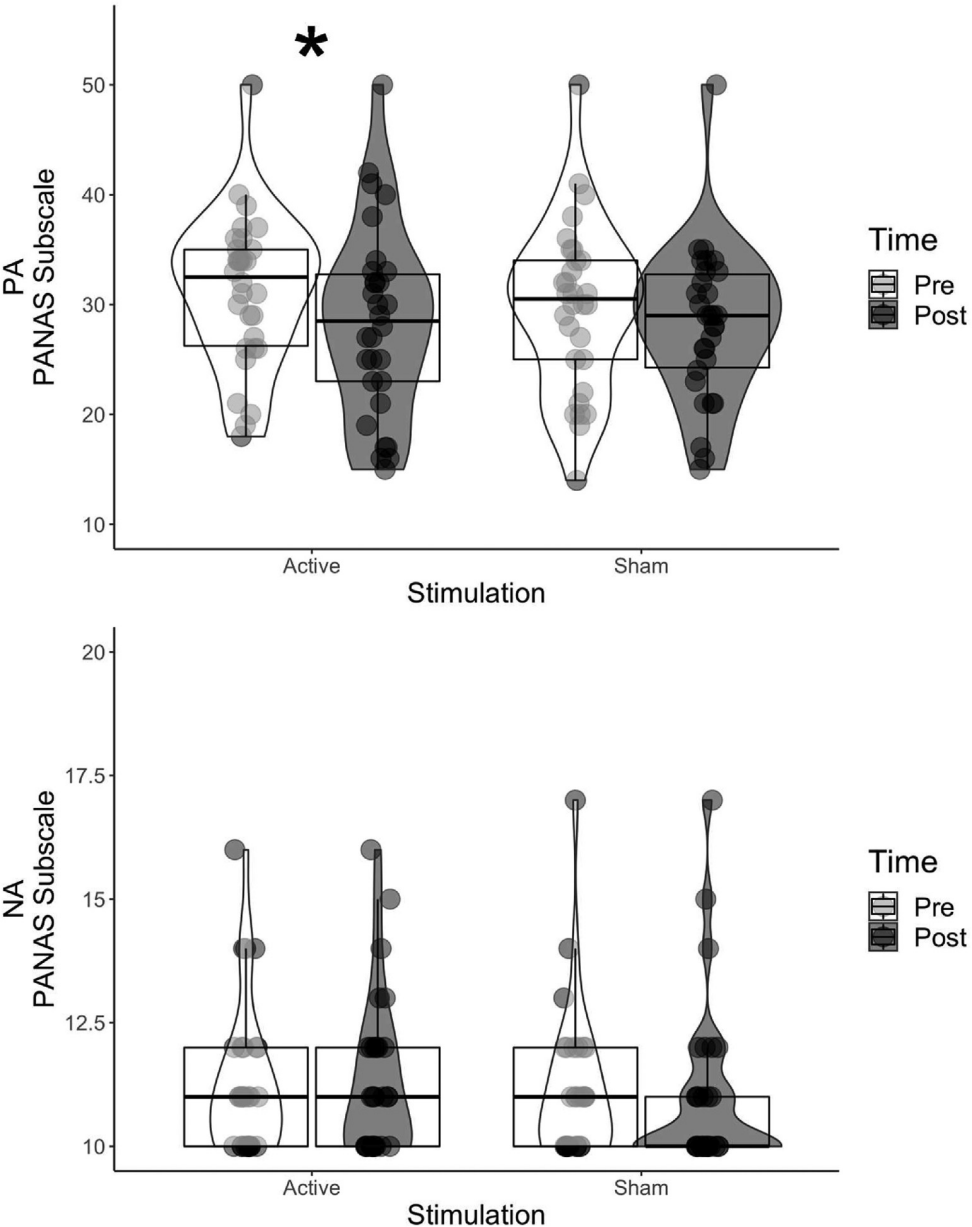
Graph depicting Positive (top, PA) and Negative Affect (bottom, NA) pre and post active and sham stimulation separately. In the exploratory analysis PA was significantly reduced post active stimulation, all other effects were non-significant.

The frequentist analysis, comprised of a paired *t*-test for post PA for active compared to post sham PA ratings. There was no significant difference following post active (*M* = 28.367; *SE* = 1.572) compared to post sham (*M* = 28.200; *SE* = 1.278) stimulation, *t*_(29)_ = .171, *p* = .865, *d* = .031 (see Fig. 5, upper panel).

##### 3.3.1.1 Exploratory analyses

The Bayesian analysis above (3.3.1) reduced the data into a 1 degree of freedom test, as pre-specified subtracting both time and stimulation factors. However, this effectively double-baselined the data, by subtracting the factors of time and stimulation, and so changes of interest could have been masked through these subtractions. We therefore, broke down the 1df analysis and performed exploratory analyses for PANAS PA, for active and sham stimulation (post-pre stimulation) separately. Using the t-distribution prior of Chaves et al., (2017) and a non-directional test for PA, we calculated the BFs for positive affect for both active (post–pre stimulation) and sham stimulation separately. For PA following active stimulation the BF = 3.694, with our sample (*M* = −2.900, *SE* = .993), suggesting positive affect reduced from pre to post active stimulation. For sham stimulation (*M* = −1.567, *SE* = .898), a reduction was also present but much smaller and the corresponding BF favoured the null, BF = .361. This suggests the small change in the sham condition may have reduced the sensitivity of the 1df test to the overall reduction in positive affect following the intervention, but this exploratory demonstration, we emphasis, does require further substantiation.

We also conducted the equivalent set of two one sample *t*-tests for the BF analyses above (post minus pre stimulation) for active and sham stimulation, separately. Congruent with the BF analyses above, active stimulation reduced positive affect, as rated on the PANAS PA, *t*_(29)_ = −2.919, *p* = .007, *d* = −.533 but sham did so only marginally *t*_(29)_ = −1.745, *p* = .092, *d* = −.319.

#### 3.3.2 Negative affect

We computed a BF for the NA subscale of the PANAS. A BF of = .037 was calculated based upon the prior t-distribution model of Chaves et al., (2017), and the observed data (*M* = .167, and *SE* = .390). This result suggests substantial support in favour of the null, indicating that TMS stimulation does not reduce negative mood, as measured on the PANAS 10.13039/501100011632NA. Similarly, for our complimentary paired-samples *t*-test (post active NA compared to post sham NA), there were no significant differences between conditions, *t*_(29)_ = 1.041, *p* = .307, *d* = .190, following active (*M* = 11.300; *SE* = .292) or sham (*M* = 11.000; *SE* = .307) stimulation for the NA subscale of the PANAS (See Fig. 4).

##### 3.3.2.1 Exploratory analysis

Following visual inspection of the data (see Fig. 5, lower panel), it became apparent that the sensitivity of the NA scale is limited by floor effects. This was confirmed on closer inspection where 45% of PANAS NA measures where at the lowest possible value, thus surpassing previously published criteria detection of floor effects on the PANAS NA at 15% of responses at the lowest value (Díaz-García et al., 2020). This suggests participants rarely associated the more negative terms of PANAS with their mood, as has been previously reported for non-clinical populations (Crawford & Henry, 2004; Mcallister, et al. 2017).

As with analysis 3.3.1 we explored analysis 3.3.2 separating the constituent contrasts of the primary registered test. We performed exploratory analyses for NA, as measured on the NA for active and sham stimulation (post-pre stimulation) separately. NA was not appreciably changed following stimulation, for active BF = .028 (*M* = .033, *SE* = .313) or sham, BF = .033 (*M* = −.133, *SE* = .338). These results suggest substantial support for the null, indicating an absence of effects on the PANAS 10.13039/501100011632NA scale, although this result is tainted by the floor effects. The equivalent one sample *t*-tests were non-significant: active *t*_(29)_ = .107, *p* = .916, *d* = .019 and sham: *t*_(29)_ = −.394, *p* = .696, *d* = −.072.

### 3.4 Replications of interest

As pre-specified, we reduced Ahn et al., (2013)'s primary interaction (Simulation × Block), to a 1df test, by computing the difference between active and sham for each block, then subjecting these scores to a linear contrast (described in the analysis section 2.7.3). The resultant BF was .243, which indicates evidence towards the null hypothesis. Congruent with Ahn et al., (2013)'s design and main analysis, we also ran a 2 × 3 frequentist Repeated Measures ANOVA, with post stimulation (active, sham) and PLT Block (1,2,3) as the within subjects' factors. No data violated the assumption of sphericity, all *ps* > .605. As predicted, there was a main effect of block for the PLT *F*_(2, 58)_ = 4.808, *p* = .012, *η*^*2*^ = .142, *d* = .81. Post-hoc comparisons using Bonferroni correction indicated that response bias for block 3 (*M* = .208; *SE* = .084) was larger than the response bias in block 1 (*M* = −.105; *SE* = .079), *p* = .019, suggesting response bias had increased irrespective of stimulation type across the blocks (see Fig. 5 post-stimulation values). This indicates the task operated as intended, where the participant's reward responsiveness bias increased over exposure to the rewarded stimulus. There were no other significant main effects or interactions either for stimulation (active versus sham) or for stimulation × block respectively, *F*_(1, 29)_ = 2.331, *p* = .138, *η*^*2*^ = .074, *d* = .57; *F*_(2, 58)_ = .433, *p* = .650, *η*^*2*^ = .015, *d* = .25.

To test the replicability of Duprat et al., 2016b, Duprat et al., 2016a main interaction (Time × Stimulation × Block with the added covariate of hedonic capacity; TEPS-CON) we computed the corresponding one-degree of freedom test, using the values reported by Duprat et al., 2016b, Duprat et al., 2016a (*M*_*diff*_ = .14, *SE* = .07, *SD* = .33). We obtained *M*_*diff*_ = -.039, and *SE* = .052 from the interaction in our study, resulting in a BF of .178, which provides strong evidence towards the null, H0. For our complimentary pre-registered frequentist statistics in line with Duprat et al., 2016b, Duprat et al., 2016a's main analyses, we conducted a 2 (time: pre, post) × 3 (block: 1,2,3) × 2 (Stimulation: active, sham) Repeated Measures ANCOVA with TEPS-CON included as the covariate. There were no significant main effects or significant interactions, all *F*s between .038 and .660, all *P* values between .423 and .963, thus indicating the introduction of the covariate did not reveal a positive effect of TMS on the outcome measures. The data did not violate the assumption of sphericity, all *P* values > .259.

### 3.5 Pre-registered: manipulation checks

We conducted a frequentist one-sample *t*-test for the average RB in block 3 of the post sham stimulation PLT. The test indicates that RB has increased compared to zero, indicating the task has been effective, with participants displaying a propensity to acquire a response bias, that is responding more to rich compared to lean stimuli over the blocks *t*_(29)_ = 2.279, *p* = .030, *η*^*2*^ = .042, *d* = .416.

Similarly, when conducting the manipulation checks modeled with the prior parameters of Ahn et al., (2013), with our observed data (*M* = .264, *SE* = .116 for post sham stimulation block 3, as pre-specified), we obtained a BF of 4.563. We also obtained a BF of 3.958 for H1 modeled upon Duprat et al., 2016b, Duprat et al., 2016a, and our observed data. These BFs suggest evidence in favour of H1- that is participants acquired a RB over the blocks.

## 4 Discussion

We examined whether active compared to sham rTMS, applied to the DLPFC, was effective in increasing reward responsiveness, in healthy participants, akin to the studies we were replicating (e.g., Ahn et al., 2013; Duprat et al., 2016b, Duprat et al., 2016a). Overall, we found no conclusive evidence for the primary hypothesis replicating the effect of active stimulation on reward responsiveness. However, we found evidence favouring the null in our replication of the interactions of interest reported by Ahn et al., (2013) and Duprat et al., 2016b, Duprat et al., 2016a. For our secondary hypotheses, we noted substantial and strong evidence towards the null for negative and positive mood, respectively, following TMS stimulation. In an exploratory break down of these omnibus tests, for negative mood, we noted substantial evidence towards the null for both active and sham stimulation conditions. Importantly, and in contrast to our predictions, positive mood was significantly reduced following active stimulation, indicating active stimulation adversely affected positive mood. We discuss these findings and their possible implications to the treatment of depression, below.

### 4.1 Primary effect of interest

Firstly, our primary hypothesis predicted that response bias would increase as a function of active stimulation in block 1 of the PLT. However, the mean difference between conditions (active vs. sham stimulation) was in the opposite direction and the corresponding Bayes Factor was inconclusive (BF = .696), indicating no conclusive support for either the null or alternative hypothesis. Here our primary 1df was insensitive but the mean difference in the opposite direction motivated an exploratory analysis which favoured the absence of a positive effect, the opposite to Ahn et al., (2013)'s finding. This finding is important as elevated reward responsiveness following repetitive TMS is used to support clinical efficacy (e.g., Ahn et al., 2013; Duprat et al., 2016b, Duprat et al., 2016a), but our failure to replicate this basic effect, suggests that such effects if real, are likely to be much smaller than previously thought and therefore of questionable clinical relevance. This is also in line with a more recent study by Duprat et al., (2017) who also failed to demonstrate an increase in reward responsiveness on the PLT following active stimulation in a sample of patients with depression. Taken together these non-significant, or null findings around elevated RB following TMS invites further questions around the clinical efficacy and utility of TMS, as endorsed by NICE (2015) and the FDA (2008).

### 4.2 Replications of interest

Ahn et al.'s (2013) primary finding, used to justify their positive conclusions, was an interaction between stimulation × block where our replication favoured the null, BF = .243. However, the theoretical underpinning of this interaction appears questionable. Ahn et al., (2013) compared post active to post sham stimulation focusing the analysis on block 1 of the PLT only, which the authors argue is evidence for the enhanced effect of one session of active TMS. This is surprising as the reward responsiveness literature commonly describes increased RB across sequential blocks of the task, as a function of reinforcement learning, not over a single block (e.g., Lancaster et al., 2012; Pizzagalli et al., 2005a, Pizzagalli et al., 2005b; Pizzagalli et al., 2008). That is participants learn to favour the most frequently rewarded stimulus, over several blocks, rather than suddenly increasing RB over a single block as described by Ahn et al., (2013). Additionally, Ahn et al., (2013) showed no evidence of a linear increase in response bias across blocks (for active stimulation), which is a typical finding across studies (Pizzagalli et al., 2008; Pizzagalli et al., 2008b, Pizzagalli et al., 2005). However, we note that a linear increase in response bias across blocks is not present in every study (e.g., Pizzagalli et al., 2008). Conversely, our study exhibited learning across the sequential blocks of the PLT, irrespective of stimulation type, indicating reward learning had occurred in line with the PLT evidence-base (e.g., Barr et al., 2008; Lancaster et al., 2012; Pizzagalli et al., 2008a, Pizzagalli et al., 2008b, Pizzagalli et al., 2008c; Pizzagalli et al., 2005a, Pizzagalli et al., 2005b).

We also found strong evidence towards the null (BF = .178) for Duprat et al., 2016b, Duprat et al., 2016a's main significant interaction (Time × Stimulation × Block with the added covariate of consummatory pleasure) and no support for the alternative hypothesis using our frequentist ANCOVA test. Duprat et al., 2016b, Duprat et al., 2016a suggested that this interaction showed iTBS increased reward responsiveness more for those participants with higher baseline hedonic capacity, as measured on the consummatory subscale of the TEPS (Gard et al., 2006). However, this finding is intriguing as theoretically we would expect the anticipatory as opposed to the consummatory elements of the TEPS to express the effects of iTBS, as the anticipatory subscale measures reward responsiveness. This inconsistency could be seen as indicative of a serendipitous result. Moreover, Duprat et al., 2016b, Duprat et al., 2016a reported that they did not correct for multiple comparisons (of which there were six ANCOVAs, yielding one marginally significant effect), again suggesting a serendipitous result.

Given that there are theoretical questions around both Ahn et al., (2013) and Duprat et al., 2016b, Duprat et al., 2016a's primary findings, the use of manipulation checks to see whether the task worked would have been useful, which were not utilised in either study. Indeed, a strength of our replication study was the inclusion of such checks indicating the PLT and reinforcement learning had occurred.

### 4.3 Secondary hypotheses: mood ratings

Our replication study did not find overall effects on critical omnibus tests, but did find the measure of positive mood decreased, opposing the prediction and becoming less positive following active TMS, under exploratory analysis. Reduced positive affect on the PANAS has been linked to anhedonia in depressed patients (Watson & Clark, 1984) and is indicative of reduced motivation and pleasure (Crawford & Henry, 2004). Similar to our finding, some early studies have reported decreased happiness in healthy controls, using self-report mood measures following TMS (George et al., 1996.; Martin et al., 1997; Pascual-Leone et al., 1996). Nevertheless, this finding was surprising, as we had expected active TMS to increase pleasure, as demonstrated by an increase in reward responsiveness on the behavioural PLT task, where contrary to our expectation active stimulation decreased positive mood. As this unexpected finding is suggestive of a detrimental effect on mental wellbeing, in the context of a treatment used to improve depressive symptoms, we advocate further confirmatory research in this area. It is worth noting however, that the levels of withdrawal due to discomfort in our experiment were relatively high (six participants compared to none reported by either Ahn et al., (2013) or Duprat et al., 2016b, Duprat et al., 2016a). Repetitive TMS applied to the DLPFC often causes activation of the muscles and nerves such as the trigeminal nerve, resulting in facial twitches that participants can find uncomfortable. Therefore, it is possible to speculate that such, largely unavoidable, discomfort could also drive the reduction of positive mood effects we observed–indeed, our experimental protocol saw the PANAS being administered almost immediately after stimulation, and thus the discomfort could have a strong influence on ratings. Consistent with our interpretation we note the reduction of positive mood was only observed for active and not sham stimulation. Here further research, including further post experiment monitoring, may be required to test the possibility that factors such as discomfort could drive effects of rTMS.

If the reduced positive mood following stimulation from the exploratory analyses were to be confirmed it would lead to a more fundamental question about whether TMS should be used as a treatment for depression, given the core premise of TMS treatment protocols for depression is to increase positive mood rather than decrease it. However, it is also worth noting that individual differences in responsiveness to TMS may be factors in the discrepancy of results between ours and both Ahn et al., (2013) and Duprat et al., 2016b, Duprat et al., 2016a's studies. A possible future avenue for TMS protocols could be measuring individual differences in stress hormones, such as cortisol, which are thought to modulate the effectiveness of TMS in non-depressed healthy control participants (e.g., Baeken et al., 2014) and in depressed patients (Baeken et al., 2009). Using personalised TMS protocols, which take into account individual differences in neural connectivity and variability (e.g., Singh et al., 2019; Caeyenberghs et al., 2018; Tik et al., 2018) could enhance the effectiveness of TMS treatments and indeed enhance reward sensitivity.

We also predicted that negative mood, as measured on the PANAS NA, would decrease as a function of TMS, that is participants would experience less negative mood following active rTMS. We found substantial support for the null hypothesis of no change following both active and sham stimulation in line with previous evidence (e.g., Baeken et al., 2008; Jenkins et al., 2002; Moulier et al., 2016; Mousimann et al., 2000). However, the absence of differences here could be driven by floor effects which limit the utility of the PANAS NA in non-clinical populations (Crawford & Henry, 2004). Prior to participation, participants underwent safety screening which excluded participants taking psychoactive medication, including antidepressants, therefore the non-clinical status of our participants is likely to have contributed to the presence of such floor effects in our data. Also, when conducting brain stimulation experiments, we aim to ensure participants are comfortable and informed. These factors may have also contributed to the prevalence of floor effects, and we would expect them to be present in any similar TMS experiment.

In light of stimulation not decreasing negative mood, and active stimulation potentially decreasing positive mood, we question the basic application of TMS to treat depression.

### 4.4 Limitations

A possible limitation of our study could be our stimulator not being identical to that of Duprat et al., 2016b, Duprat et al., 2016a. This resulted in one of the components of the theta burst protocol being generated at a slightly different frequency to those of Duprat (i.e the mean frequency of the triplet bursts was 45.967 Hz as opposed to 50 Hz, See Supplementary Material for a full breakdown of frequency used, https://osf.io/sep4g). However, our protocol was still facilitatory, with frequencies well above ∼5 Hz, above which are postulated to induce excitatory effects (Huang et al., 2005; Siebner & Rothwell, 2003), and still above the high frequency TMS protocol used by Ahn et al., (2013). As Ahn et al., (2013) used a different stimulator, and a different high frequency TMS protocol rather than iTBS such as Duprat et al., 2016b, Duprat et al., 2016a, we argue that the differences between Ahn et al., (2013) and Duprat et al., 2016b, Duprat et al., 2016a replication are greater than the iTBS frequency differences between our replication and that of Duprat et al., 2016b, Duprat et al., 2016a, and the stimulator we used is far more commonly used and therefore of greater clinical relevance.

## 5 Conclusions

In sum, participants in our study demonstrated reward responsiveness akin to that found in the extant PLT literature. However, we failed to replicate Ahn et al., (2013) and Duprat et al., 2016b, Duprat et al., 2016a's key findings that active stimulation would positively modulate reward responsiveness. Nevertheless, we found, in an exploratory analysis, that positive affect was reduced, akin to anhedonia, following active stimulation, which is concerning for the use of TMS as a treatment for depression as such TMS is thought to reduce anhedonia. We also found support towards the null for all other key effects, including decrease of negative affect, which highlights the need for further research to parse what depressive symptomatology may be targeted effectively via TMS, if not negative mood, or anhedonia. These fundamental questions are pivotal for the continued safe and ethical roll-out of TMS to treat depression.

## Credit author statement

**Lowri Hadden** – Conceptualization; Data curation; Formal analysis; Investigation; Methodology; Funding acquisition; Validation; Visualization; Project administration; Recruitment & Data Collection; Writing – original draft; Writing – review & editing.

**Helen Penny** – Conceptualization; Funding acquisition; Supervision; Writing – review & editing.

**Adam M. Partridge** – Recruitment; Data Collection; Validation; Resources; Writing – review & editing.

**Alex L. Jones** – Resources; Visualization; Writing – review & editing.

**Thomas M. Lancaster** – Formal analysis; Data curation.

**Christopher Allen** - Conceptualization; Validation; Resources; Recruitment & Data Collection; Data curation; Formal analysis; Investigation; Methodology; Project administration; Funding acquisition; Formal analysis; Resources; Supervision; Writing – original draft; Writing – review & editing.

## Open practices

The study in this article earned Open Data, Open Materials and Preregistered badges for transparent practices. Materials for the study are available at: https://osf.io/724gt/

## Footnotes

1 This sentence was added for clarity and transparency during the stage 2 review process to enhance precise information about the timing of the PLT, and stimulation.

2 This sentence was edited during the stage 2 review to enhance clarity.

3 We have modified this sentence since the stage 1 registered report, removing the sentence “but they do not give precise information about how they located this area” to highlight how Ahn et al., (2013) identified the DLPFC.

4 Due to the differences between Duprat et al., 2016a, Duprat et al., 2016b and Ahn et al., (2013)'s protocols and our attempts to reconcile these differences in the most clinically informative way, strictly speaking this study should not be interpreted as a ‘direct’ or ‘exact’ replication of either study but an amalgamation of both studies.

5 Clarification of the contrast was added after stage 1 following a reviewer's comment.
