# Supplementary material for: Pre-frontal stimulation does not reliably increase reward responsiveness
Source: Cortex. 2023 Feb;159:268–85. doi: 10.1016/j.cortex.2022.11.011 (PMC10823575; doi:10.1016/j.cortex.2022.11.011)
Supplement: Multimedia component 1 [file mmc1.docx]

**Supplementary Material**

Summary of frequency drop-off relating to the 50Hz component of the theta burst stimulation, which decreased in frequency at stimulator intensities greater than 50% of maximum (Magstim Rapid^2^).

| **PID** | **Motor Threshold**  **(MT)** | **Theoretical intensity (1.1 x MT)** | **Frequency** | **Frequency Drop-off** | |  |  |  |  |
| --- | --- | --- | --- | --- | --- | --- | --- | --- | --- |
| 1 | 53 | 58.3 | 46Hz | 4Hz | |  |  |  |  |
| 2 | 46 | 50.6 | 50Hz | 0Hz | |  |  |  |  |
| 3 | 65 | 71.50 | 39Hz | 11Hz | |  |  |  |  |
| 4 | 50 | 55 | 48Hz | 2Hz | |  |  |  |  |
| 5 | 53 | 58.3 | 46Hz | 4Hz | |  |  |  |  |
| 6 | 44 | 48.4 | 50Hz | 0Hz | |  |  |  |  |
| 7 | 48 | 52.8 | 49Hz | 1Hz | |  |  |  |  |
| 8 | 55 | 60.5 | 45Hz | 5Hz | |  |  |  |  |
| 9 | 54 | 59.4 | 45Hz | 5Hz | |  |  |  |  |
| 10 | 66 | 72.6 | 39Hz | 11Hz | |  |  |  |  |
| 11 | 65 | 71.50 | 39Hz | 11Hz | |  |  |  |  |
| 12 | 44 | 48.40 | 50Hz | 0Hz | |  |  |  |  |
| 13 | 55 | 60.5 | 45Hz | 5Hz | |  |  |  |  |
| 14 | 60 | 66 | 42Hz | 8Hz | |  |  |  |  |
| 15 | 61 | 67.1 | 42Hz | 8Hz | |  |  |  |  |
| 16 | 65 | 71.50 | 39Hz | 11Hz | |  |  |  |  |
| 17 | 57 | 62.7 | 44Hz | 6Hz | |  |  |  |  |
| 18 | 48 | 52.80 | 49Hz | 1Hz | |  |  |  |  |
| 19 | 54 | 59.40 | 46Hz | 4Hz | |  |  |  |  |
| 20 | 49 | 53.90 | 48Hz | 2Hz | |  |  |  |  |
| 21 | 54 | 59.4 | 46Hz | 4Hz | |  |  |  |  |
| 22 | 47 | 51.7 | 49Hz | 1Hz | |  |  |  |  |
| 23 | 46 | 50.60 | 50Hz | 0Hz | |  |  |  |  |
| 24 | 55 | 60.50 | 45Hz | 5Hz | |  |  |  |  |
| 25 | 59 | 64.9 | 43Hz | 7Hz | |  |  |  |  |
| 26 | 40 | 44 | 50Hz | 0Hz | |  |  |  |  |
| 27 | 41 | 45.10 | 50Hz | 0Hz | |  |  |  |  |
| 28 | 48 | 52.80 | 49Hz | 1Hz | |  |  |  |  |
| 29 | 46 | 50.6 | 49Hz | 1Hz | |  |  |  |  |
| 30 | 52 | 57.2 | 47Hz | 3Hz | |  |  |  |  |
|  |  |  |  |  | |  |  |  |  |
| **Mean** | **52.667** | **57.933** | **45.967 Hz** | **4.033 Hz** | |  |  |  |  |
|  |  |  |  |  | |  |  |  |  |
| PID= participant Identification; MT= Motor Threshold | | | | |  | |  |  |  |
